# Indents

**Published:** 1902-09-15

**Authors:** 


### INDENTS.

**☞ Brief Articles of Technical or Scientific Value will receive notice and comment. Contributions invited.**

Hardening Plaster Models.—Dissolve boric acid in warm water and add sufficient ammonia to form a borate of ammonia, which remains in solution. The models can be bathed in this solution or it may be added when mixing the plaster.—*Medical News*.

Competition and Ethics.—The desire and effort to compete with the low fees of the advertiser, and poor workmen, by men who want to be ethecal, is a dangerous tendency, so far as professional culture and standing is concerned.—G. Good, in *Cosmos*.

Antidote for Formaldehyde.—A convenient and effective antidote for formaldehyde is ammonia water, a few drops well diluted or the aromatic tinctur in water. Formalin and its preparations are being so largely used now by dentists that it may be will to remember this.

To Clean Hypodermic Needles.—Boil in a solution of carbonate of soda for ten minutes—Doin, in *Bulletin of General Therapeutics*. A better way is to boil in a two per cent solution of lysol and with a sterile hot air syringe blow the water out of the needle while it is hot.

Polishing Amalgam Filling.—It is just as essential that amalgam filling should be polished, as that the cavity should be properly shaped and its border properly prepared for the joint. Too many forget or rather neglect this valuable incident of practice. --Dr. E. K. Wedelstaedt, in *Dental Summary*.

Separating and Treatment.—As a preliminary to separating the teeth with mechanical separators, spray the gums with a solution of cocain by compressed air. After separating, crowd the teeth together again, and allay any irritation by an application of iodine and tincture of myrrh.—H. Hofheinz, in *Cosmos*.

To Introduce Varnishes and chloropercha into a cavity without touching the borders, use a small probe with a fan shaped bulb on the end. This will take up and hold the liquid without allowing it to run down the shank and will readily part with it when brought into contact with the tooth substance.—S. J. Spence.

Oxyphosphate Cement does not corrode the pulp. —Dr. G. V. Black in the *Items of Interest* maintains that zinc oxyphosphate cements are not generally responsible for the destruction of pulps. He believe that where a pulp dies under an oxyphosphate filling, it is due either to lack of judgment in selecting a situable case, or to faulty manipulation.

To Sharpen Engine Burs.--Use a Ruby Gem disk, made in Worcester, Mass., about one inch in diameter thin and cone shaped, with the base of cone mounted toward the hand piece. Hold the bur between thumb and index finger of left hand and slowly rotate and trace out each blade. With the disk run rapidly by the electric engine preferably. --W. S. G. Elliot, in *Items*.

INJECTING A PULP.—The pulp was inaccessible to the hypodromic point. The cavity was filled with gutta percha except a small opening into which was fitted a conical plug of soft rubber through which the needle was introduced. By holding the soft rubber in tight with a forked instrument, the cocaine solution can be readily forced into the pulp. Dr. Kimball.—*International Journal.*

CONSCIENTIOUSNESS.—Of all the qualities for which we may have a reputation, there is nothing more noble, more unselfish, more honest, than to be a conscientious dentist. Our conscientiousness may not always find its just reward with our patients, but it never fails to win us two principal compensations—thee steem of our *confreres*, and above all, self-respect.—Dr. Wm. Hirshfield, in *Cosmos*.

DISKING ENAMEL MARGIN.—Dr. Lewis in the *Cosmos* says that sand paper disks on enamel margins preparatory to filling produce a margin to which it is impossible to perfectly adept gold.—This may be true if care is not exercised to run the disk true and at right angles to the surface of the tooth. If properly used a more perfect marging can be secured than with a chisel or other device.—ED.

PACKING MAT GOLD.—Dr. N. S. Hoff made some test fillings in cavities of uniform size in a metal block to show that mat gold should be as carefully condensed as any other preparation of gold to get thorough welding and sufficient strength to resist the stress of mastication. He also showed the advantage of combining mat gold with, pellets in order to get additional strength and close adaptation.

Obtunding Dentin.—Take equal parts of pulverized nitrate of silver and eucaine and make into a paste with carbolic acid. Dry the cavity and saturate it with carbolic acid, and place the paste in the cavity and cover it with soft gutta percha. A second application is sometimes necessary, but usual the tooth may be cut without much inconvenience to the patient.—E. H. Raymond, in *International Journal.*

A new Anesthetic Acoine is the name of an interesting product which is destined to oust cocaine, morphine, chloral, antipyrene and other anesthetics. A little pinch dropped into the cavity of an aching tooth is said to instantly banish pain. Acoine's properties were recently reported to the French Academy of Medicine by Dr. Chauvel, and are based on divers experiments. Acoine is said to have the great advantage of being non-toxic.

German Dentists Working Against Americans.—According to newspaper reports, the native dentists of Germany are asking the physicians' societies to secure the passage of a bill prohibiting German medical men from administering anesthetics when the "mechanical dentists," as the German practitioners style the Americans, are pulling teeth. Physicians, however, are not favorable to the move, perceiving it to be spite-work and not justifiable. —*Digest.*

Sunday Dentistry Prohibited.—Pittsburg has passed blue laws which, among other things, prohibit dentists from doing any unnecessary work on Sunday. Some of the rabit Puritans want the laws to read that dentists shall do no work at all, and that even the hospitals shall be closed, but the greater majority of residents, as well as the dentists, are complaining against such strictness. The question of what is "necessary" work will be interesting to solve.—*Digest.*

Antidote for Cocain.—Gelsemium is recommended by an exchange as a useful antidote for cocain poisoning. It has a depressant effect on the nervous system and may be of some worth to allay nervous excitement of cocain. It has no marked effect upon the heart but stimulates the respiratory function, which it destroys in poisonous doses. It does not have sufficiently marked influence in counteracting cocain to afford any positive statement as to its effects.

Inlay Impressions.—A new impression compound called "Impression Lac," is just on the market by Ash & Sons. It is not unlike the high grade sealing wax used on ladies stationary. It softens best by dry heat, over a bunsen or alcohol flame. It can be kneaded until fit to use and should be removed before it gets quite hard. A number of useful claims are made for it: such as supporting loosend teeth, steadying clamps and separators, bites and impressions for crown work, etc.

Dentist and Public.—The ethical relations of the dentist to the public is entirely a question of honesty. He should always give his best service regardless of the fee received. It is not a breach of professional ethics either for a young practitioner to charge less fees than a more experienced one exacts, providing he does not use that fact to divert patients from the more experienced practitioner. The young man's skill may be equal from a technical standpoint, but his judgement can not have a corresponding value.—Dr. Hofheinz, in *Cosmos.*

Born with teeth.—An item going the rounds of the Maine press to the effect that a babe recently born at Calais, to Mr. and Mrs. W. A. Petersen, had at birth a full-size lower front tooth, which is considered remarkable, recalls a similar case in this town some years ago, when a worthy couple were presented with twin boys, each of whom had a number of teeth when born, as many residents can affirm, and which their little tombstone in the city cemetry also confirms, bearing this remarkable inscription: "Twins of Mr. and Mrs. ——, born with teeth."—*Eastport, Me., despatch to Boston Record.*

Separating Fluid.—

| | |
|---|---|
| ℞. Shell-lac . . . . . . . . . . . | ʒ xij |
| Borax . . . . . . . . . . . . . | ʒ vj |
| Water . . . . . . . . . . . . | ℥ x |

Mix and keep in a warm—not hot—place until solution is complete, then add

| | |
|---|---|
| Sugar . . . . . . . . . . . . | ʒ x |
| Glycerine . . . . . . . . . . . | ʒ vj |
| Water q. s. ad. . . . . . . . | ℥ xvj |

Shake well until sugar dissolves, then let it stand a few days, and decant the clear liquid into a wide mouth bottle. If desirable some water soluable die may be added to color. This solution will penetrate the plaster impression without obliterating its details, and leave a nice gloss upon the surface.—*Dental Era.*

To retain a matrix.—Dr. Crouse says, take a quick setting cement and place it about the copper matrix which has been adjusted to the tooth, and in five minutes it will admit of malleting without becoming disturbed. Dr. McLain, in the *International Journal*, intimates that the unyielding oxyphosphate is undiserable, as it will not allow a perfect adaptation of the gold at the cavity margins. He suggests that the matrix be tied to the tooth by several turns of well waxed ligature, as this will allow the matrix to give away from the tooth enough to let the gold crowd out beyond the margins.

Professional Diplomacy.—More patients are lost through faulty tact in address, than by lack of skill in mechanical or operative technique. As financial success goes, an agreeable presence is more potent than skill or brains ; yes, more powerful than both combined. It is not wise to be brusque, and any attempt at "blarneying" will be detected by the better class of patients. One must not be too business like, or the patient will believe you are practicing for money only ; and the laity love to see a man sacrifice himself "for love of his profession." It is difficult for many to "be all things to all men," as the apostle was, yet herein lies the secret of success.—*Clippings*.

Relative Value of Anesthetics.—The danger rate begins to increase after the thirtieth year under chloroform, but with ether it remains constant until after the fiftieth year. Apparently none of the mixtures of ether or chloroform gives such satisfactory results as ether alone. Nitrous oxid with or without oxygen shows the lowest percentage of complications. The final conclusion of the anesthetic committee of the British Medical Association is that the most important factor is the skill of the anesthetist, and that to insure the best results he must be one of large experience and good judgment.—*Ed. Albany Medical Annals.*

Acetlylene Gas Explosion.—Dr. Griffis, of Paris, Texas, was quite severely injured by the unexpected explosion of an acetlylene gas generator of his own devising which he was exhibiting at the recent meeting of the Texas Dental Association. A similar explosion of a gasoline generator used in heating a furnace for baking porcelain is reported also from San Francisco. While the manufacturers of these appliances assure us of their absolute safety, we are never sure that there may not be somewhere a leak which results disastrously when not expected. The moral is that in using all such highly volatile substances, the greatest care should always be taken to prevent such accidents.

Mallets.—Time was when we heard a great deal of hand pressure, but what we want is a uniform density of the filling from beginning to end. I usually use two automatic mallets for mat gold, although I have all the approved mallets made. This reminds me of a clinic some twenty-five years ago, Dr. McWilliams put in a gold filling for Dr. McMillan which I believe is still preserving the tooth. The Doctor wanted a mallet and none was to be had, so he stooped down and slipped off one of his low cut shoes, and I malleted that filling in with the heel of his shoe. It was a good mallet. The form of mallet is not so important as the fact that it is effectively used in condensing the mat gold. Dr. Hetrick, in *Western Journal.*

Porcelain Inlays.—Dr. W. J. Reeves, of Chicago, demonstrated his method of constructing a porcelain inlay and the setting of it. Dr. Reeves uses platinum for the matrix and high fusing bodies. He made a beautifully matched and fitting inlay for a patient restoring a lost corner of an incisor. He does not prepare the cavity with undercuts, and consequently can adapt and remove the inlay matrix readily. He uses Harvard cement mixed thin to set the madrix and does not groove the inlay to get a secure attachment for the cement, but etches that side of the inlay with hydrofluoric acid, and depends on the close adaptation for an unusual hardening of the cement and its permanent retention. He claims the inlays will hold more securely if closely adapted, and the cement will be less liable to dissolve out.

Filtered Water Dangerous.—Dr. A. L. Wood says that the domestic filter is a dangerous article of the worst description. People rely upon it in fancied security, while in ninety-nine cases out of every one hundred the water is more dangerous to health and life after passing through it than before. All soluble mineral salts and all impurities of every description, including the deadly poisons from disease germs, which are held in solution, pass through the very best filter at all times, as freely as the water itself, and, unless the filter is cleaned and sterilized several times a day, which is rarely if ever done, the germs of typhoid fever and other diseases multiply with great rapidity within the filter itself and pass through with the water. Many eminent chemists and scientists have testified to the truth of these statements.—*Dict. and Hyg. Gaz.*

INFLAMMATION AT APEX AFTER DEVITALIZATION.—A few weeks ago I anesthetized by pressure and removed four pulps (from four persons) with vapocain, followed by carbolic acid combined with chloroform. I also used suprarenal by pressure on the anesthetized pulps to check the beeding. Filled at the same sitting. In each case I had severe apical inflammation, lasting from two to five days. Was this caused by the carbolic acid, or the suprarenal, or was it that one or both of these drugs, by checking the flow of the blood, prevented the escape of the cocain, or what was it? The inflammatory symptoms above described may fairly be attributed to the use of vapocain (an ethereal solution of cocain). One of the effects of cocain is to produce capillary contraction, followed by relaxation and more or less congestion. Had the severed vessels been allowed to bleed freely, the congestion would have been relieved. The suprarenal extract, by checking hemorrhage, prevented this, and the pulp-canals being immediately filled with an impermeable material, there was no escape for oozing serum—pressure and inflammation being the result. If the adrenal extract was not perfectly aseptic, and it very readily undergoes putrefaction, septic poisoning from that source may have been an additional complication.—*Brief*.

